# Maternal and fetal cardiometabolic recovery following ultrasound-guided high-intensity focused ultrasound placental vascular occlusion

**DOI:** 10.1098/rsif.2019.0013

**Published:** 2019-05-01

**Authors:** Caroline J. Shaw, Ian Rivens, John Civale, Kimberley J. Botting, Beth J. Allison, Kirsty L. Brain, Y. Niu, Gail ter Haar, Dino A. Giussani, Christoph C. Lees

**Affiliations:** 1 Department of Physiology, Development and Neuroscience, University of Cambridge, Cambridge CB2 3EG, UK; 2 Institute of Reproductive and Developmental Biology, Imperial College London, London W12 0HS, UK; 3 Joint Department of Physics, Institute of Cancer Research, Sutton SM2 5NG, UK; 4 Cardiovascular Strategic Research Initiative, University of Cambridge, Cambridge, UK; 5 Department of Obstetrics and Gynaecology, University Hospitals Leuven, 3000 Leuven, Belgium

**Keywords:** high-intensity focused ultrasound, placental vasculature, selective vascular occlusion, pregnancy

## Abstract

High-intensity focused ultrasound (HIFU) is a non-invasive method of selective placental vascular occlusion, providing a potential therapy for conditions such as twin–twin transfusion syndrome. In order to translate this technique into human studies, evidence of prolonged fetal recovery and maintenance of a healthy fetal physiology following exposure to HIFU is essential. At 116 ± 2 days gestation, 12 pregnant ewes were assigned to control (*n* = 6) or HIFU vascular occlusion (*n* = 6) groups and anaesthetized. Placental blood vessels were identified using colour Doppler ultrasound; HIFU-mediated vascular occlusion was performed through intact maternal skin (1.66 MHz, 5 s duration, *in situ* I_SPTA_ 1.8–3.9 kW cm^−2^). Unidentifiable colour Doppler signals in targeted vessels following HIFU exposure denoted successful occlusion. Ewes and fetuses were then surgically instrumented with vascular catheters and transonic flow probes and recovered from anaesthesia. A custom-made wireless data acquisition system, which records continuous maternal and fetal cardiovascular data, and daily blood sampling were used to assess wellbeing for 20 days, followed by post-mortem examination. Based on a comparison of pre- and post-treatment colour Doppler imaging, 100% (36/36) of placental vessels were occluded following HIFU, and occlusion persisted for 20 days. All fetuses survived. No differences in maternal or fetal blood pressure, heart rate, heart rate variability, metabolic status or oxygenation were observed between treatment groups. There was evidence of normal fetal maturation and no evidence of chronic fetal stress. There were no maternal injuries and no placental vascular haemorrhage. There was both a uterine and fetal burn, which did not result in any obstetric or fetal complications. This study demonstrates normal long-term recovery of fetal sheep from exposure to HIFU-mediated placental vascular occlusion and underlines the potential of HIFU as a potential non-invasive therapy in human pregnancy.

## 1. Introduction

There has been increasing interest in the use of high-intensity focused ultrasound (HIFU) in fetal medicine since it has the potential to target fetal tissue and blood vessels within the amniotic cavity, and placental blood vessels without requiring highly intrusive access to the uterus. A number of applications have been suggested, including the treatment of broncho-pulmonary sequestration and sacrococcygeal teratoma [1]. Recently, HIFU has been used to ablate the soft tissue of the umbilical cord insertion point in the acardiac twin, using both B-mode and colour Doppler ultrasound imaging for targeting, in pregnancies complicated by twin reversed arterial perfusion sequence with a 50% efficacy rate [2,3].

Ultrasound-guided HIFU has also been proposed as a method of selective vascular occlusion [1], potentially providing a non-invasive treatment for conditions such as twin–twin transfusion syndrome (TTTS), which arise from abnormal placental vascular anastomoses [4,5]. This addresses an important unmet clinical need, as TTTS remains the leading cause of death, disability and premature delivery in twins, and invasive therapies such as selective fetoscopic laser ablation of placental anastomoses offer no survival advantage to affected fetuses compared to amniodrainage [6]. This is probably due more to the invasive nature of fetoscopic techniques, which require entry into the intrauterine space (hence disrupting the amniotic sac), than to the occlusion of the abnormal placental vascular connections which cause the condition. HIFU offers a non-invasive alternative to such techniques. HIFU-mediated ablation of *in utero* soft tissue, including placental tissue, has been demonstrated in human and animal studies [2,4,7–9]. Our group has demonstrated selective placental vascular occlusion in animal studies, both transuterine and through intact maternal skin [5,10]. While providing evidence of feasibility, these studies did not constitute sufficient ‘proof of principle’ to translate our techniques into human studies. In our earlier transuterine study, the protocol did not seek to demonstrate fetal survival beyond 30 min from the end of exposure to HIFU in sheep pregnancy. Prior to translation into human therapy, evidence of prolonged fetal recovery, as well as the long-term maintenance of a healthy fetal physiological state following HIFU treatment, is essential [5].

Our second study using pregnant sheep demonstrated that it is possible to use ultrasound-guided HIFU to occlude placental vasculature selectively through intact maternal skin, although 50% of subjects experienced a skin burn, and a further 33% experienced skin erythema. This raises the additional question of whether sufficient HIFU energy could be delivered to the intrauterine space through maternal intact skin without incurring skin damage [10], prior to translation to human clinical therapy.

We have now developed a wireless data acquisition system (CamDAS: Cambridge Data Acquisition System) that permits maternal and fetal cardiovascular data to be recorded continually from the end of surgical instrumentation, through recovery from anaesthesia and longitudinally until the end of the experiment in free-moving animals [11]. Therefore, the primary aim of this current study was to establish fetal and maternal cardiovascular and metabolic long-term recovery using CamDAS in sheep following ultrasound-guided HIFU applied through intact maternal skin to occlude placental blood vessels selectively. The secondary aim was to determine the rates of successful vascular occlusion and associated iatrogenic harm when lower ultrasound energy levels were delivered to the target blood vessels.

## 2. Material and methods

All procedures were performed in accordance with the UK Animals (Scientific Procedures) Act 1986 and were approved by the Ethical Review Committee of the University of Cambridge.

Twelve singleton Welsh mountain sheep fetuses at 116 ± 2 d gestational age (median ± range, term approx. 147 days) were used. Six ewes were exposed to HIFU and six animals were not, forming the control group.

### 2.1. Induction and maintenance of general anaesthesia

Animals were fasted, with free access to water, for 24 h before the induction of anaesthesia. The maternal abdomen was shaved 24–48 h prior to the experimental procedures. On the day of exposure to HIFU or sham (day 0), general anaesthesia was induced with alfaxalone 3 mg kg^−1^ (IV, Alfaxan, Jurox) and maintained with isoflurane (inhaled, 1.5–2.5% in 4 : 1 O_2_:N_2_O). Ewes were mechanically ventilated and maternal oxygen saturation and end-tidal carbon dioxide (EtCO_2_) were monitored non-invasively; maternal SpO_2_ was maintained greater than 94% and EtCO_2_ was maintained at less than 6%.

### 2.2. Preparation of maternal abdominal skin and establishment of an acoustic window

Following induction of anaesthesia, maternal abdominal skin was washed with iodine scrub solution (Vetasept: 7.5% povidone iodine, Animalcare Ltd) to remove dirt and lanolin and washed again with plain water. If proceeding to HIFU exposures, remaining hair was chemically depilated (Nair^®^ hair removal cream, Church & Dwight Co.), the skin was washed with plain water, then wetted with degassed water. A ‘water bag’ made from polythene was placed in contact with the maternal abdomen, filled with degassed water and suspended from a fixed support. Trapped air between the plastic and the skin was removed by smoothing the plastic against the skin, until no remaining air pockets were identified visually. The water in the bag was continuously cooled to 20.5–25.7°C and degassed to 1.9–3.0 mg l^−1^ dissolved oxygen throughout the duration of the HIFU protocol.

### 2.3. High-intensity focused ultrasound protocol

Following anaesthesia and skin preparation, but prior to any invasive surgical procedures, HIFU was applied through intact maternal skin. The HIFU transducer (Sonic Concepts H148MR transducer, frequency 1.66 MHz, 64 mm diameter, 63 mm focal length, 19 mm central aperture for ultrasound imaging, focal spot diameter 1.2 mm, axial focal spot length 8.9 mm) was supported in its position within the water bag using a gantry arm with three-dimensional automated positioning (able to move on *X*-, *Y*- and *Z*-axes). Manual angulation of the transducer was possible before the start of an exposure series, but not during one (electronic supplementary material, figure S2). A signal generator fed a power amplifier (A330, E&I Ltd, Rochester, NY) which was used to control the transducer output. A graphical user interface (Matlab R2013a, Mathworks) on a laptop computer was used to control and log (i) the automated gantry position, (ii) signal generator settings and (iii) timing of exposures. B-mode and colour Doppler imaging were used to identify target vessels (4–10 MHz sector phased array diagnostic ultrasound transducer, P10–4, Z.one Zonare, Mindray, China). The diagnostic transducer was centrally mounted behind the HIFU transducer in a fixed position and provided imaging through the central aperture. The target vessels were chosen to be within 1–4 cm below the skin and able to be imaged satisfactorily by colour Doppler. There were six target vessels in each animal in the HIFU-exposed group, and none in the control animals.

Four to seven HIFU exposures of 5 s duration, spaced 5 s and 2 mm apart, were applied. This separation was chosen to give a contiguous lesion, in the form of a single linear track, placed across the vessel. The number of exposures was selected based on the length of this track, which was required to start and end in placental tissue. The acoustic power used was designed to deliver approximately 2.5–3.0 kW cm^−2^ estimated *in situ* intensity to the target vessel at different focal depths beneath the maternal skin: greater than or equal to 30.0 mm = 78 W; 25.0–29.9 mm = 62 W; 20.0–24.9 mm = 50 W; less than or equal to 14.9 mm = 40 W. Estimated *in situ* intensity was calculated based on the attenuation coefficients of the overlying animal tissues, determined in a previous study [12]. The safety precautions used were that: (i) movement of the mechanical gantry arms was restricted to a single direction along an axis at 90° to the direction of sound/vertical midline of the ultrasound image and (ii) HIFU exposure series were not planned or delivered in vascular targets in which fetal parts could be seen in the post-focal region.

Target vessels were assessed before, and immediately after, HIFU exposure using colour Doppler in the same three-dimensional position (determined using the automated gantry), with 3 s video clips being recorded. Treatment success was defined as no flow being detectable on colour Doppler after treatment using the lowest velocity scale setting (−6.3 to 6.3 cm s^−1^), and the highest gain setting that did not cause colour saturation. In the event of a failed occlusion, one further HIFU exposure track per target vessel was allowed, unless the maternal injury was suspected after the first track. The maternal skin was inspected for evidence of skin changes and photographed before and after the completion of each series of HIFU exposures. Superficial skin reddening which blanched with pressure and which resolved fully at any time within 24 h was described as erythema; more severe thermal skin injuries were described as a skin burn.

### 2.4. Maternal and fetal surgery

Following the completion of HIFU exposures, or, for the control group following the induction of anaesthesia, surgery was commenced under aseptic conditions as described previously [5]. In brief, a midline incision was made in the maternal abdomen to access the pregnant uterus. The rectus sheath, omentum and serosal surface of the uterus were visually examined for evidence of thermal injury. A hysterotomy was performed in the uterus and the fetal hindlimbs were exteriorized, with care taken to preserve amniotic fluid. On one side, the femoral artery was cannulated with an arterial catheter. On the contralateral side, a 2 mm aperture time-transit time flow probe (R series, Transonic Systems Inc., Ithaca, USA) was placed around the femoral artery. A third catheter was secured to the fetal hind limb for the monitoring of the amniotic cavity pressure. The maternal left femoral artery and vein were also cannulated. Incisions in the fetal skin, uterus, rectus sheath and maternal abdominal skin were closed. Vascular catheters were exteriorized via a keyhole incision on the maternal flank; flow probe cables were exteriorized through the contralateral flank.

Antibiotics were given following uterine closure (600 mg benzylpenicillin IA, Crystapen, Genus Pharmaceuticals, Newbury, UK). Inhalational anaesthesia was withdrawn and the animal was extubated when the spontaneous respiratory effort was re-established.

### 2.5. Post-operative care

Ewes were housed in individual floor pens with a 12 L : 12 D cycle with *ad libitum* access to hay, nuts and water. Maternal antibiotics (procaine benzylpenicillin 15 mg kg^−1^ IM, Depocillin^®^, Intervet UK Ltd, Milton Keynes, UK) were administered for 5 days following surgery and fetal antibiotics (600 mg benzylpenicillin IA, Crystapen, Genus Pharmaceuticals, Newbury, UK) were administered daily until the end of the experiment. Maternal analgesia (carprofen 1.4 mg kg^−1^ SC, Rimadyl^™^, Pfizer Ltd, Sandwich, UK) was administered for three post-operative days, and as required if signs of maternal pain were evident based on behaviour, facial expressions, stance and feeding patterns. Animals were checked daily for signs of maternal pain and distress, problems with mobility, bladder or bowel function, preterm labour or rupture of membranes or vaginal bleeding.

### 2.6. Assessment of materno-fetal cardiovascular status during recovery

Intra-arterial catheters were connected to pressure transducers (ArgoTrans^™^ Transducer, Argon Medical Devices Inc., Plano, TX, USA) positioned at the level of the maternal heart. Phasic arterial pressure recordings were corrected for atmospheric pressure (maternal) or amniotic cavity pressure (fetal). Pressure transducers and time-transit transducers were connected to a customized data acquisition system, the CamDAS [11,13]. This is an ambulatory system, housed in a protective jacket worn by the pregnant ewe. It samples (at 500 KHz) and continuously transmits digitized arterial blood pressure and arterial flow information to a laptop running IDEEQ (Maastricht Instruments, Maastricht, Netherlands). Recorded data were available thereafter for offline analysis.

Data were consolidated into files containing 24 h (00:00:00 to 23:25:59) and uploaded into a data analysis program (Labchart 7 Pro, AD Instruments Ltd, New Zealand). Mean values for sequential 1-min epochs (minute means) were generated for the systolic peak, diastolic nadir and amplitude of maternal and fetal arterial blood pressures. Minute means for mean arterial flow rate and mean amniotic cavity pressure were also generated by this method. Minute means for each parameter were exported to a customized spreadsheet (Excel^®^, Microsoft Corporation, Redmond, WA, USA) and logic checked for artefactual data which were non-physiological and produced due to catheter blockage or equipment error, which was excluded. From the remaining data, minute mean values within any given hour were then averaged to give hourly means, and hourly means were further averaged to give the daily means reported here (electronic supplementary material, figure S1). This method of using time epochs and sequential averaging is recognized to prevent time periods with greater periods of excluded ‘artefact data’ being under-represented in the overall average [14].

### 2.7. Assessment of materno-fetal metabolic status during recovery

Blood samples taken from the maternal and fetal descending aortic catheters (0.3 ml each) were drawn into sterile syringes on a daily basis (electronic supplementary material, figure S1). These were used to determine pH, arterial base excess (ABE), concentration of bicarbonate, partial pressures of oxygen and carbon dioxide (ABL5 Blood Gas Analyser, Radiometer, Copenhagen, Denmark). Blood glucose and lactate concentrations were obtained from an automated analyser (YSI 2300 Stat Plus, Yellow Springs Instruments, Ohio, USA). In the HIFU-exposed animals, haemoglobin and oxygen saturation of the blood were determined using an ABL80 Flex analyser (Radiometer, Copenhagen, Denmark). In control animals, the haemoglobin and oxygen saturation of blood were determined using an OSM3 analyser (Radiometer, Copenhagen, Denmark), due to a change of laboratory equipment during the duration of the experiments. Results obtained by these two methods have been previously compared internally at time of machine replacement and are highly comparable (private communication). Values for haematocrit were obtained in duplicate using a microhaematocrit centrifuge (Hawksley, Lancing, UK).

On the fifth day post-HIFU (baseline) and the day of post-mortem (day 20, electronic supplementary material, figure S1), a further 5 ml of maternal and fetal blood was collected into EDTA and centrifuged (5000 rpm, 4°C, 5 min) for plasma extraction. All samples were then frozen at −80°C for later analysis. The concentration of fetal cortisol in plasma was quantified using a commercially available cortisol indirect enzyme-linked immunosorbent assay kit (RE52061, IBL International, Hamburg, Germany), as described in detail recently [15]. In brief, duplicate 20 µl plasma aliquots (undiluted, previously unthawed) were tested according to the product instructions. The optical density of the wells was read (wavelength 450 nm, ELx800 Absorbance Reader, Biotek, Winooski, VT, USA) within 10 min of the end of the assay. A standard curve for interpretation of the results of each plate was produced by plotting the mean optical density of manufacturer-provided standard concentrations of cortisol against their known concentrations. The equation of the resulting line of best fit was used to extrapolate cortisol concentrations from measured optical densities of fetal samples. The inter-assay coefficient of variation (CV) was 5.1%; the intra-assay CV was 4.8%.

### 2.8. Assessment of fetal heart rate variability during recovery

The fetal heart rate was sampled daily in six, 5-min blocks spaced evenly between 00.00 and 06.00 using the waveform derived from fetal descending aorta catheter signals, from the first post-operative day to the day of the post-mortem examination, using a method previously described by our group [15]. Blocks were visually identified as quiet sleep or active sleep. Time domain and power spectral analysis were then performed in Labchart; based on the previously published literature, the frequency boundaries used were: very low frequency 0–0.04 Hz, low frequency (LF) 0.04–0.15 Hz, high frequency (HF) 0.15–0.4 Hz [16–18]. Mean daily values for the standard deviation of normal to normal R–R intervals (SDNN), root mean square of the successive differences, absolute and normalized LF and HF, LF/HF ratio and total power were calculated daily from the 5-min samples. Short-term variation (STV) was calculated using R–R intervals generated by Labchart in a customized Excel spreadsheet, as previously published [19].

### 2.9. Post-mortem examination

On ‘day 20’, the ewe and fetus were euthanized for post-mortem examination and tissue collection under schedule one of the UK Animals (Scientific Procedures) Act 1986. A slow IV injection into the maternal jugular vein of 120 mg kg^−1^ pentobarbitone sodium (Pentoject^®^, Animalcare Ltd, York, UK) was used for this purpose. A systematic visual inspection of (i) maternal skin, (ii) maternal rectus sheath, (iii) anterior and posterior surface of the uterus, (iv) fetal skin (dorsal, ventral, left and right lateral views) and (iv) maternal bladder and bowel was performed. Fetuses were weighed and measured. All placentomes were excised, bisected and examined for evidence of tissue damage, in both exposed and control animals. Placentomes with evidence of tissue damage were retained for histological examination. Collected tissues were immersion fixed in 10% neutral buffered formalin for 5 days before embedding in paraffin wax. Eight micrometre sections were stained with haematoxylin and eosin (H&E) to provide a detailed view of damaged and undamaged tissues, and with phosphotungstic acid–haematoxylin (PTAH) stains to identify areas of fibrin deposition. Measurements taken from histological sections were not corrected for shrinkage, as it was not known whether shrinkage rates of damaged and undamaged tissue were comparable. Measurements therefore represent a minimum size, which may have been reduced artificially by fixation.

### 2.10. Statistical analyses

The study was powered to detect a difference in means of greater than or equal to 2.5 at *α* = 0.05 with a power of 80%, based on the published data of response to an acute insult (hypoxia or hypotension) in chronically instrumented sheep fetuses.

All statistical analysis was performed in SPSS (v. 22, IBM, NY, USA). Graphs were drawn in GraphPad Prism (v. 6, GraphPad Software, Inc., San Diego, CA, USA). Statistical significance was accepted when *p* < 0.05 for all tests, although where applicable individual *p*-values are presented in graphs, tables and text.

Continuous data were assessed for normality using the Shapiro–Wilk test. Descriptive analysis was performed using mean ± standard error of the mean (s.e.m.), unless otherwise stated. A two-tailed Student's *t*-test was used to compare means (normally distributed) or a Mann–Whitney *U*-test was used to compare medians (non-parametric data).

To assess the effect of HIFU treatment on a continuous dependent variable when additional independent variables needed to be considered, two-way ANOVA testing was used. A repeated measures two-way ANOVA was used to investigate the change in the dependent categorical variable due to the interaction of gestational age/time with HIFU treatment. If a significant effect or interaction was identified, *post hoc* testing was performed as applicable to identify the source of variation. Values were compared to a baseline set on day 5 post-operative. This was done as standard experimental practice is to allow 4 days post-operative recovery in the pregnant sheep model and perform baseline recordings on day 5.

## 3. Results

### 3.1. Efficacy of vascular occlusion

**Figure 1.**
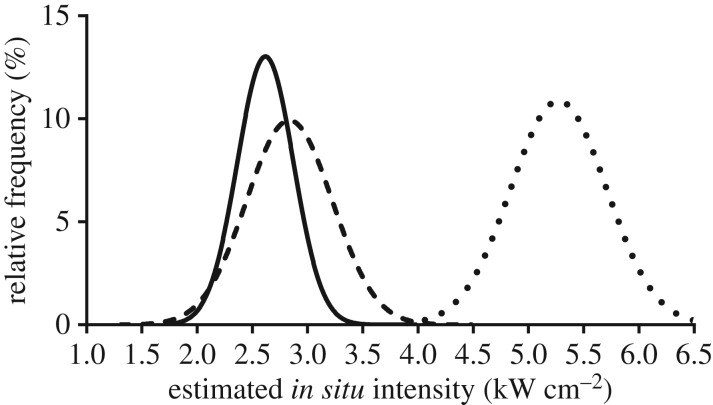
De-escalation of estimated *in situ* intensities resulting in successful occlusion. The curves show the distribution of relative frequencies of estimated *in situ* intensities resulting in successful occlusion in this animal group (solid line), the previous transabdominal animal group (dashed line (9)), and the transuterine animal group (dotted line (4)). In this animal group, the median estimated *in situ* intensity required to produce occlusion was 2.6 kW cm^−2^ (IQR 2.5–2.9, range 1.8–3.9). In the previous transabdominal group, it was 2.9 kW cm^−2^ (IQR 2.5–3.1, range 2.3–4.4) and in the transuterine group, it was 5.4 kW cm^−2^ (IQR 5.0–5.5, range 3.9–5.6). This represents a deliberate de-escalation of *in situ* intensities delivered in recognition that the targets in the transuterine animal group were exposed to *in situ* intensities in excess of what was required to produce vascular occlusion.

Based on the comparison of pre- and post-HIFU exposure colour Doppler imaging, 36 of 36 target placental blood vessels were successfully occluded. Regarding successful occlusions, 33 of 36 vessels were occluded following the initial HIFU exposure series. In the remaining three vessels, targeting errors of between 5 and 10 mm were identified during the experimental protocol. Once these errors had been corrected, these remaining three vessels were successfully occluded. The median estimated *in situ* intensity which resulted in successful occlusion was 2.6 kW cm^−2^ (interquartile range 2.5–2.9 kW cm^−2^, range 1.8–3.9 kW cm^−2^). The distribution of estimated *in situ* intensities which resulted in successful occlusion is shown in figure 1, which also presents the equivalent data from our previous transdermal and transuterine studies for comparison [5,10]. The mean depth of the target vessels was 24 mm (s.d. ± 6 mm) beneath the maternal abdominal skin surface.

**Figure 2.**
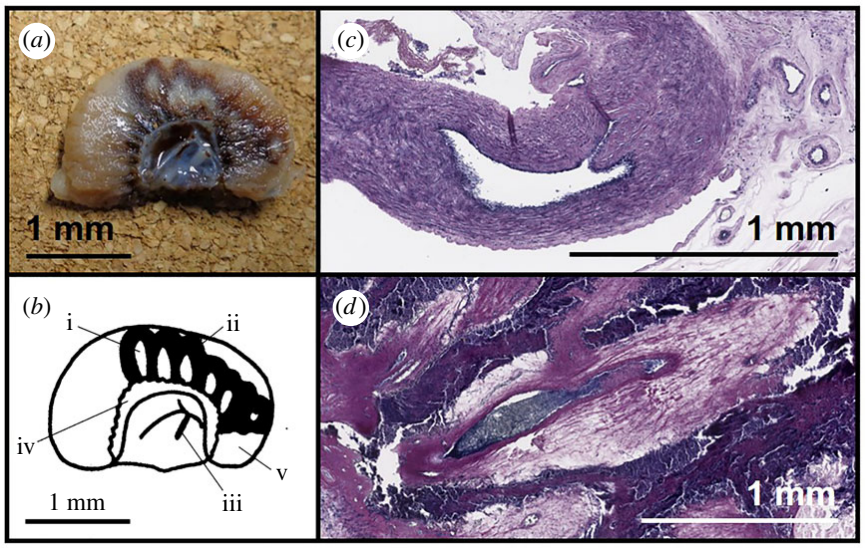
Macroscopic tissue damage and histological confirmation of vascular occlusion after 21 days. (*a*) The photograph shows a placentome bisected 21 days after exposure to HIFU, photographed after formalin fixation for 24 h, with areas of tissue pallor surrounded by an area of tissue darkening. (*b*) The line diagrams of the photograph in (*a*) represent (i) tissue pallor, (ii) tissue darkening, (iii) placental vessels, (iv) area of materno-fetal exchange surrounding the origin of placental vessels, (v) placentome soft tissue. (*c*) PTAH stain of fetal vessels in an unexposed placentome. The lumen is open and unobstructed and the connective tissue around the vessels has regular purple staining of normal collagen. (*d*) PTAH stain of a fetal vessel exposed to HIFU and thought to be occluded by colour flow Doppler studies. The lumen is stained black, indicating that it is occluded by organized fibrin, a definitive appearance of the occlusive clot. Surrounding the vessels there are regions of irregular, pale staining in the collagenous regions suggestive of vacuolar degeneration. (Online version in colour.)

At post-mortem examination, 21 days following exposure to HIFU, six placentomes with macroscopic evidence of HIFU tissue damage were recovered from each animal in the HIFU-exposed group (a total of 36, figure 2*a*). There was no evidence of haemorrhage of placental vessels, nor damage to the fetal membranes, at the time of post-mortem examination (blood or clots in the allantoic membranes). No macroscopic tissue damage was seen in any of the placentomes of the control animals.

Trapped erythrocytes within the vessel lumen, suggestive of the occlusive clot, were found in 36/36 placentomes, which had been exposed to HIFU under histological examination using H&E staining. PTAH staining confirmed the presence of organized fibrin in those vessels in which trapped erythrocytes were seen (figure 2*b*). Vacuolar change, suggestive of degeneration of the connective tissue surrounding vessels, was found surrounding occluded vessels in 27/36 placentomes (figure 2*c*). The mean diameter of occluded vessels was 1.4 mm (range 0.3–4.1 mm, without correction for tissue shrinkage).

### 3.2. Fetal survival and growth

All the fetuses survived, undelivered, to the end of the 21-day follow-up period. Fetal weight at post-mortem examination was 3.3 ± 0.2 kg in the control group, and 3.4 ± 0.1 kg in the HIFU-exposed group (*p* = 0.58). Fetal body mass index was 17.3 ± 0.8 kg m^−2^ in control fetuses and 16.4 ± 0.7 kg m^−2^ in those HIFU-exposed (*p* = 0.46). Both control and HIFU-exposed fetuses showed appropriate symmetric growth, as the ratio of bi-parietal diameter to abdominal circumference was normal and similar between groups 0.18 ± 0.01 (control) and 0.21 ± 0.01 (HIFU, *p* = 0.06).

### 3.3. Fetal cardiovascular recovery

**Figure 3.**
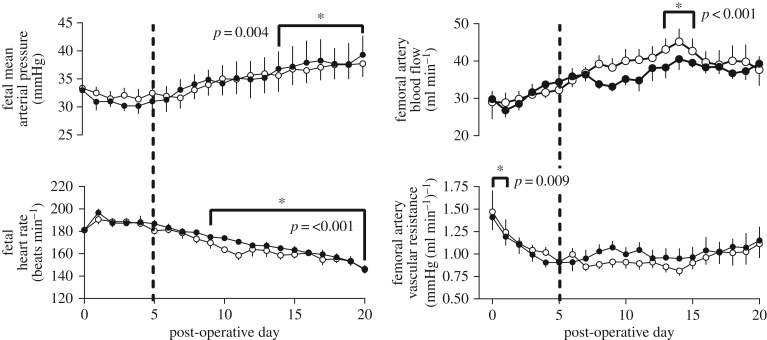
Fetal mean arterial pressure, heart rate, femoral arterial blood flow and vascular resistance. Values represent the mean ± s.e.m. of fetal mean arterial blood pressure, heart rate, fetal femoral arterial blood flow and vascular resistance averaged over sequential 24 h periods in HIFU-exposed (filled circles; *n* = 6) or control (open circles; *n* = 6) animals. The dashed line represents the day 5 baseline against which other values were compared. Statistical significance was assessed using a repeated measures two-way ANOVA with *post hoc* Tukey's test; * denotes a significant difference of time with respect to baseline, with exact *p*-values given on the graphs. There was no significant effect of treatment.

Following 5 days of post-operative recovery, fetal heart rate, fetal mean arterial blood pressure, fetal femoral arterial blood flow and fetal femoral arterial vascular resistance were the same between HIFU-exposed and control groups. Fetal femoral vascular resistance was elevated in the first 24 h following surgery and gradually recovered to basal levels by the fourth post-operative day, without evidence of an additive effect of HIFU exposure. In all fetuses, there was an ontogenic increase in arterial blood pressure and femoral blood flow and a fall in fetal heart rate with advancing gestation. These maturational effects of advancing gestational age on fetal cardiovascular function were not affected by HIFU exposure (figure 3).

### 3.4. Fetal metabolic recovery

Immediately following the reversal of anaesthesia (day 0, table 1), fetuses in both groups showed evidence of a partially buffered mixed respiratory and metabolic acidosis, due to the elevated arterial partial pressure of carbon dioxide (P_a_CO_2_) and arterial lactate concentration. In the fetuses exposed to HIFU, the ABE was reduced compared to baseline, while the arterial bicarbonate concentration (HCO^−^_3_) remained unchanged compared to baseline. In the control fetuses, ABE was elevated compared to the baseline, and HCO^−^_3_ remained normal. The ABE and HCO^−^_3_ were significantly different between treatment groups.

**Table 1.** Fetal metabolic status during recovery. Values represent the mean ± s.e.m. of fetal arterial oxygen partial pressure (P_a_O_2_), oxyhaemoglobin saturation, haemoglobin, haematocrit, pH, ABE, carbon dioxide partial pressure (P_a_CO_2_), concentration of lactate, bicarbonate and glucose in the descending aorta and femoral arterial oxygen and glucose delivery for animals in the HIFU-exposed (*n* = 6) and control (*n* = 6) groups. Values for days 0–5 in HIFU-exposed or control fetuses (post-operative recovery) are presented as daily means; values for the ‘monitoring period’ are presented as the mean ± s.e.m. of 5 days' values. Before values given in the ‘monitoring period’ were grouped into 5-day means they were compared individually to each other and the baseline. Grouping them into 5-day means did not alter any significant outcomes (or lack thereof); they are presented grouped for ease of presentation. Significant differences in the effect of time and treatment group were assessed using a repeated measures two-way ANOVA. Significance was accepted as *p* < 0.05. Where authorized, *post hoc* Tukey's and Sidak's tests were used to demonstrate a significant effect of time (*) or the effect of treatment (†), respectively, during the experimental protocol, with relevant *p* numbers given in the table.

variable	treatment group	post-operative recovery (day)					baseline	monitoring period (day)			*p-*value	
		0	1	2	3	4	day 5	6–10	11–15	16–20	(*time)	(^†^treatment)
pH	HIFU	7.24 ± 0.01*	7.36 ± 0.02	7.33 ± 0.01	7.33 ± 0.02	7.34 ± 0.02	7.33 ± 0.01	7.33 ± 0.02	7.34 ± 0.02	7.35 ± 0.02	<0.001	0.13
	control	7.23 ± 0.02*	7.39 ± 0.01	7.37 ± 0.01	7.36 ± 0.01	7.36 ± 0.01	7.37 ± 0.01	7.33 ± 0.04	7.36 ± 0.01	7.37 ± 0.01		
P_a_CO_2_ (mmHg)	HIFU	59 ± 1^†^*	45 ± 2	48 ± 2	48 ± 1.3	49 ± 1	48 ± 1	49.8 ± 0.5	49.3 ± 0.4	50.0 ± 0.6	<0.001	0.04
	control	64 ± 1^†^*	51 ± 1	47 ± 2	52 ± 1.5	50 ± 1	52 ± 1	51.7 ± 0.9	53.8 ± 0.9	54.3 ± 0.8		
ABE (mmol l^−1^)	HIFU	−3.2 ± 1.4^†^*	−1.8 ± 0.8^†^	−1.6 ± 0.7^†^	−2.2 ± 0.6^†^	−0.6 ± 1.6	−0.6 ± 0.8	−1.1 ± 0.6	−0.8 ± 0.6	0.9 ± 0.5	0.04	0.04
	control	4.6 ± 0.7^†^*	3.4 ± 0.9^†^	1.2 ± 1.0^†^	2.2 ± 1.6^†^	1.6 ± 1.0	1.6 ± 0.3	−0.4 ± 0.7	1.7 ± 0.5	2.5 ± 0.3		
bicarbonate (mEq l^−1^)	HIFU	23.6 ± 1.2^†^	22.8 ± 1.1^†^	23.4 ± 1.0^†^	22.4 ± 1.1^†^	24.4 ± 1.8	23.8 ± 0.7	24.1 ± 0.5	24.3 ± 0.5	25.5 ± 0.6	0.34	0.03
	control	26.6 ± 0.4^†^	28.0 ± 0.7^†^	27.4 ± 1.0^†^	27.0 ± 1.7^†^	26.4 ± 1.0	26.2 ± 0.6	24.8 ± 0.7	26.1 ± 0.7	25.9 ± 0.7		
arterial lactate (mmol l^−1^)	HIFU	2.0 ± 0.2*	1.3 ± 0.1*	1.0 ± 0.1	1.0 ± 0.1	1.0 ± 0.2	0.9 ± 0.1	1.0 ± 0.1	1.0 ± 0.1	1.0 ± 0.1	<0.001	0.29
	control	2.1 ± 0.4*	1.6 ± 0.3*	1.0 ± 0.1	1.1 ± 0.1	1.0 ± 0.1	1.0 ± 0.1	1.0 ± 0.1	1.1 ± 0.1	1.1 ± 0.1		
P_a_O_2_ (mmHg)	HIFU	20 ± 1	22 ± 1	21 ± 2	20 ± 2	19 ± 2	20 ± 2	22 ± 1	21 ± 1	20 ± 1	0.06	0.14
	control	20 ± 1	22 ± 1	21 ± 2	19 ± 1	20 ± 1	19 ± 1	20 ± 1	19 ± 1	20 ± 1		
oxyhaemoglobin saturation (%)	HIFU	57 ± 4	64 ± 2*	55 ± 5	53 ± 6	55 ± 6	58 ± 4	57 ± 2	56 ± 2	56 ± 1	<0.001	0.55
	control	61 ± 2*	65 ± 3*	57 ± 6	49 ± 4	53 ± 4	53 ± 4	54 ± 2	54 ± 2	51 ± 2		
femoral arterial oxygen delivery (mmol min^−1^)	HIFU	103 ± 10	105 ± 9	95 ± 7	91 ± 9	90 ± 9	94 ± 9	125 ± 6*	141 ± 6*	136 ± 7*	0.001	0.31
	control	92 ± 11	105 ± 11	108 ± 13	88 ± 13	102 ± 8	104 ± 3	101 ± 5	122 ± 6*	110 ± 3		
haemoglobin (g dl^−1^)	HIFU	11.1 ± 0.6*	9.9 ± 0.8*	9.1 ± 0.7	9.2 ± 0.7	8.7 ± 0.5	8.6 ± 0.8	9.5 ± 0.4	9.9 ± 0.4	10.6 ± 0.3	<0.001	0.40
	control	11.2 ± 0.6*	10.0 ± 0.5*	9.2 ± 0.6	8.4 ± 0.4	8.5 ± 0.3	8.7 ± 0.3	8.5 ± 0.3	8.8 ± 0.3	9.2 ± 0.4		
haematocrit (%)	HIFU	32.6 ± 0.6*	30.7 ± 2.4*	27.2 ± 1.1	28.2 ± 2.0	26.7 ± 1.5	26.6 ± 2.3	27.0 ± 0.5	28.1 ± 0.4	30.0 ± 0.3	<0.001	0.90
	control	33.0 ± 0.3*	31.6 ± 2.0*	28.6 ± 1.8	26.3 ± 1.5	26.5 ± 1.1	27.6 ± 1.4	26.8 ± 0.7	28.4 ± 1.1	29.2 ± 1.3		
glucose concentration (mmol l^−1^)	HIFU	0.8 ± 0.1	1.0 ± 0.1	0.8 ± 0.1	1.0 ± 0.1	0.9 ± 0.1	0.9 ± 0.1	1.0 ± 01	0.9 ± 0.1	0.8 ± 0.1	0.07	0.26
	control	0.8 ± 0.1	0.8 ± 0.1	0.8 ± 0.1	0.8 ± 0.1	0.9 ± 0.1	1.0 ± 0.2	0.8 ± 0.1	0.7 ± 0.1	0.7 ± 0.1		
femoral arterial glucose delivery (µmol min^−1^)	HIFU	20 ± 3	28 ± 5	25 ± 3	31 ± 3	30 ± 4	30 ± 4	37 ± 3*	39 ± 3*	33 ± 2	0.02	0.44
	control	20 ± 4	24 ± 5	28 ± 3	29 ± 6	32 ± 4	33 ± 4	31 ± 2	31 ± 2	29 ± 1		

By the first post-operative day, P_a_CO_2_ and arterial lactate concentration had normalized compared to baseline in both treatment groups and remained normal throughout the duration of the follow-up period. The ABE and HCO^−^_3_ had also normalized when compared to baseline by the first post-operative day, although the difference between groups due to the effect of treatment persisted until the third post-operative day, with the HIFU-exposed group having a lower ABE and HCO^−^_3_ than the control group fetuses.

At every point measured, fetal oxygenation remained normal, in terms of both oxyhaemoglobin saturation and arterial partial pressure of oxygen (P_a_O_2_), including immediately following the reversal of anaesthesia, when the fetuses were mildly acidotic (day 0, table 1). There was a transient elevation in fetal oxyhaemoglobin saturation on the first post-operative day, with no change in P_a_O_2_. There was an increase in femoral arterial oxygen delivery with increasing gestational age, with no difference between treatment groups noted.

Fetal haemoglobin and haematocrit were elevated immediately following the reversal of anaesthesia and on the first post-operative day (day 0–1, table 1). After this, both normalized and remained stable compared to the day 5 baseline, with no evidence of fetal anaemia or effect of gestational age or treatment noted.

The concentration of fetal arterial glucose and femoral arterial glucose delivery following reversal of anaesthesia (day 0, table 1) were normal when compared to baseline (day 5) and were stable throughout the follow-up period. There was no effect of gestational age or treatment group observed.

### 3.5. Fetal cortisol levels

**Figure 4.**
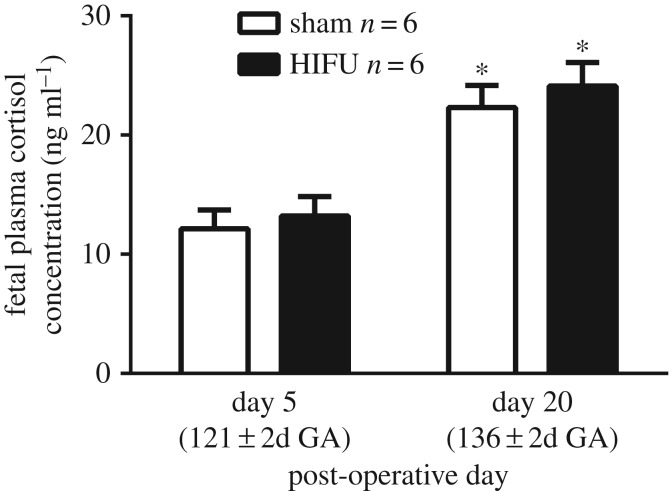
Fetal plasma cortisol levels. Values represent mean ± s.e.m. of fetal plasma cortisol concentrations 5 and 20 days after surgery in HIFU-exposed (black bar, *n* = 6) or control (open bar, *n* = 6) fetuses. Significant differences: *p < 0.001 effect of gestational age, two-way repeated measures ANOVA with Tukey's and Sidak's *post hoc* tests. There was no effect of treatment.

Fetal plasma cortisol concentrations measured under basal conditions were not different between treatment groups at either of the two time points at which they were measured, day 5 and day 20 post-operative (figure 4). At baseline (day 5), the fetal cortisol concentrations were low; however, there was a gestational age-dependent increase in plasma cortisol concentrations between day 5 and day 20, with no effect of HIFU treatment.

### 3.6. Fetal heart rate variability

**Figure 5.**
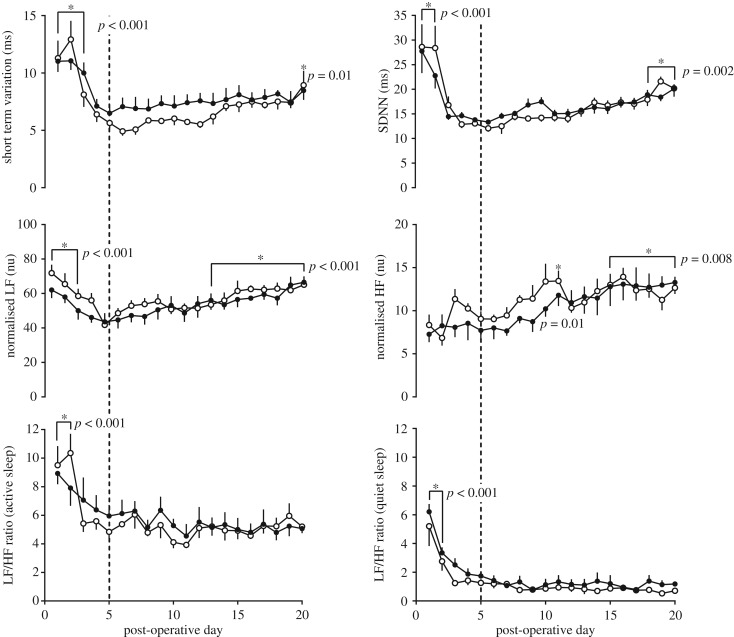
Change in FHRV indices with gestational age. Values represent mean ± s.e.m. in active sleep of short-term variation (STV), standard deviation of normal to normal intervals (SDNN), normalized LF (nLF) and normalized HF (nHF) power and LF/HF ratio, and in quiet sleep of LF/HF ratio in HIFU-exposed (filled circles; *n* = 6) or control (open circles; *n* = 6) animals. The dashed line represents the day 5 baseline against which other values were compared. Significant differences in the effect of time and treatment group were assessed using a repeated measures two-way ANOVA. Where authorized, *post hoc* Tukey's tests demonstrated a significant effect of time (*), with relevant *p* numbers given on the graphs. There was no effect of treatment group.

STV, SDNN and normalized low frequency (nLF) power in active sleep were all elevated during the first three post-operative days when compared to the day 5 baseline and had normalized by the fourth post-operative day. Normalized high frequency (nHF) power was not different compared to baseline in the first four post-operative days. The ratio of LF to HF power (LF/HF ratio) was elevated for the first two post-operative days in both active and quiet sleep and had normalized by the third post-operative day compared to day 5 of baseline. These effects were seen in both the HIFU-exposed and control fetuses, with no effect of treatment group observed. There were also gestational age-dependent increases in STV, SDNN, nLF and nHF power in active sleep. The rate and degree of these increases were not affected by treatment. There was no change in the ratio of LF to HF (LF/HF ratio) power in either quiet or active sleep states related to gestational age (figure 5).

### 3.7. Maternal cardiovascular recovery

**Figure 6.**
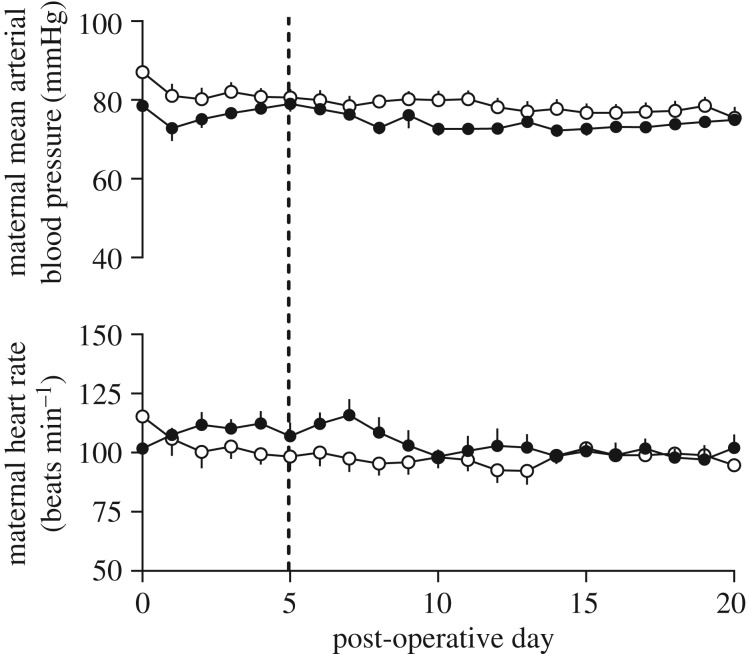
Maternal cardiovascular recovery from HIFU placental vascular occlusion. Values represent the mean ± s.e.m. of maternal mean arterial pressure and heart rate averaged over sequential 24 h intervals for HIFU (filled circles; *n* = 6) and control (open circles; *n* = 6) treatment groups, measured from pulsatile flow in the maternal descending aorta arterial catheter, and compared to the baseline established on day 5 post-operative (dotted line). No significant effects of time or treatment were found using a repeated measures two-way ANOVA.

Maternal heart rate and mean arterial blood pressure were not altered from baseline, either in the immediate post-operative period or at any point during the 21-day follow-up period. No effect of treatment was observed (figure 6).

### 3.8. Maternal metabolic recovery

Maternal P_a_O_2_, oxyhaemoglobin saturation and arterial glucose concentration were normal following the reversal of anaesthesia and remained stable in the immediate post-operative period and for the duration of the follow-up period.

However, immediately following the reversal of anaesthesia (day 0, table 2), mothers in the control group showed evidence of a respiratory acidosis, which was fully resolved by the first post-operative day. Their pH was lowered secondary to a raised P_a_CO_2_, and to a lesser degree, a raised arterial lactate concentration._._ There was evidence of partial compensation of this acidosis, with a raised ABE and raised arterial bicarbonate concentration. In the HIFU-exposed mothers, the pH was not different to baseline; however, the arterial bases excess and arterial bicarbonate were both raised, suggesting a fully compensated respiratory acidosis. In support of this, P_a_CO_2_ was raised by clinical standards compared to baseline, but this change did not achieve statistical significance. These values had normalized by the first post-operative day. There was a significant difference in both pH and P_a_CO_2_ between control and HIFU-exposed animals, with control animals showing a greater respiratory acidosis. The arterial lactate concentration was also raised in these animals compared to baseline, with no difference between treatment groups. While statistically significant, the magnitude of this elevation of arterial lactate was not of clinical consequence and was fully restored to baseline by the second post-operative day. Thereafter, maternal pH, P_a_CO_2_, ABE, arterial bicarbonate and lactate concentrations remained stable for the duration of the follow-up period.

**Table 2.** Maternal metabolic status during recovery. Values represent the mean ± s.e.m. of maternal femoral arterial oxygen partial pressure (P_a_O_2_), oxyhaemoglobin saturation, haemoglobin, pH, ABE, carbon dioxide partial pressure (P_a_CO_2_), concentration of lactate, bicarbonate and glucose in HIFU-exposed (*n* = 6) and control (*n* = 6) groups. Values for days 0–5 following HIFU or sham exposures (post-operative recovery) are presented as daily means; values for the ‘monitoring period’ are presented as the mean ± s.e.m. of 5-day values. Before values given in the ‘monitoring period’ were grouped into 5-day means they were compared individually to each other and the baseline. Grouping them into 5-day means did not alter any significant outcomes (or lack thereof); they are presented grouped for ease of presentation. Significant differences in the effect of time and treatment group were assessed using a repeated measures two-way ANOVA. No significant differences for the effect of treatment were found (†) but where authorized *post hoc* Tukey's tests demonstrated a significant effect of time (*) during the experimental protocol, with relevant *p* numbers given in the table.

variable	treatment group	post-operative recovery (day)					baseline	monitoring period (day)			*p*-value	
		0	1	2	3	4	day 5	6–10	11–15	16–20	(*time)	(^†^treatment)
pH	HIFU	7.47 ± 0.02^†^	7.47 ± 0.02	7.48 ± 0.01^†^	7.45 ± 0.02^†^	7.48 ± 0.01^†^	7.48 ± 0.01	7.46 ± 0.01	7.46 ± 0.01	7.47 ± 0.01	<0.001	0.04
	control	7.41 ± 0.01*	7.52 ± 0.03	7.55 ± 0.01	7.53 ± 0.01	7.55 ± 0.01	7.51 ± 0.01	7.49 ± 0.01	7.47 ± 0.01	7.50 ± 0.01		
ABE (mmol l^−1^)	HIFU	4.8 ± 1.4*	−2.0 ± 0.9	0.8 ± 0.6	0.8 ± 1.1	1.5 ± 0.6	1.3 ± 1.4	0.9 ± 0.7	1.8 ± 0.3	2.3 ± 0.5	0.01	0.07
	control	5.3 ± 1.2*	2.8 ± 2.0	4.0 ± 2.2	3.0 ± 0.6	4.0 ± 0.4	3.3 ± 0.3	3.0 ± 0.7	2.8 ± 0.5	3.9 ± 0.5		
P_a_CO_2_ (mmHg)	HIFU	39 ± 3^†^	31 ± 1	33 ± 1	33 ± 1	34 ± 1	34 ± 2	34 ± 1	36 ± 1	36 ± 1	<0.001	0.02
	control	50 ± 2*	30 ± 1	30 ± 2	33 ± 1	32 ± 1	32 ± 2	33 ± 1	34 ± 1	33 ± 1		
arterial lactate (mmol l^−1^)	HIFU	1.1 ± 0.2*	1.2 ± 0.3*	0.6 ± 0.1	0.7 ± 0.1	0.7 ± 0.3	0.6 ± 0.1	0.6 ± 0.1	0.5 ± 0.1	0.5 ± 0.1	<0.001	0.88
	control	1.0 ± 0.1*	1.1 ± 0.3*	1.0 ± 0.3*	0.8 ± 0.1	0.4 ± 0.1	0.3 ± 0.3	0.5 ± 0.1	0.5 ± 0.1	0.6 ± 0.1		
bicarbonate (mEq l^−1^)	HIFU	27.8 ± 1.7*	21.3 ± 0.5	23.3 ± 0.8	23.0 ± 1.3	23.8 ± 0.5	23.8 ± 1.3	23.2 ± 0.6	24.3 ± 0.4	24.9 ± 0.5	<0.001	0.13
	control	30.3 ± 1.7*	24.0 ± 2.2	25.8 ± 1.8	27.0 ± 1.2	25.2 ± 0.5	25.8 ± 0.6	25.5 ± 0.7	25.3 ± 0.6	26.2 ± 0.5		
P_a_O_2_ (mmHg)	HIFU	109 ± 19	96 ± 3	92 ± 6	92 ± 4	89 ± 3	94 ± 3	99 ± 2	93 ± 2	99 ± 1	0.09	0.10
	control	108 ± 22	111 ± 7	105 ± 6	96 ± 3	103 ± 3	100 ± 1	101 ± 3	105 ± 3	97 ± 2		
oxyhaemoglobin saturation (%)	HIFU	104 ± 1	103 ± 1	102 ± 2	101 ± 1	102 ± 1	103 ± 1	104 ± 1	103 ± 1	104 ± 1	0.71	0.61
	control	102 ± 1	103 ± 1	102 ± 1	102 ± 1	102 ± 1	103 ± 1	103 ± 1	102 ± 1	103 ± 1		
haemoglobin (g dl^−1^)	HIFU	8.4 ± 0.6*	10.9 ± 0.3	10.4 ± 0.2	10.2 ± 0.4	10.3 ± 0.5	10.4 ± 0.3	10.5 ± 0.2	10.1 ± 0.2	10.5 ± 0.3	<0.001	0.22
	control	8.9 ± 0.6*	11.2 ± 0.2	10.6 ± 0.2	10.0 ± 0.3	10.0 ± 0.7	9.6 ± 0.2	9.8 ± 0.2	9.7 ± 0.2	9.7 ± 0.1		
haematocrit (%)	HIFU	22 ± 2*	34 ± 1	32 ± 1	32 ± 1	32 ± 1	31 ± 1	32 ± 1	33 ± 1	32 ± 1	<0.001	0.34
	control	23 ± 3*	35 ± 1	32 ± 1	31 ± 1	31 ± 1	30 ± 1	30 ± 1	31 ± 1	32 ± 1		
glucose (mmol l^−1^)	HIFU	2.5 ± 0.3	2.6 ± 0.3	2.3 ± 0.1	2.5 ± 0.2	2.5 ± 0.2	2.4 ± 0.1	2.6 ± 0.1	2.5 ± 0.1	2.3 ± 0.1	0.16	0.81
	control	2.6 ± 0.1	2.5 ± 0.2	2.4 ± 0.4	2.5 ± 0.1	2.7 ± 0.1	2.6 ± 0.1	2.7 ± 0.1	2.5 ± 0.1	2.3 ± 0.1		

In both treatment groups, the mothers were haemodiluted by the intraoperative crystalloid infusions following reversal of anaesthesia, leading to an apparent fall in haemoglobin, which had self-corrected by the first post-operative day. Maternal haemoglobin and haematocrit remained stable following this and for the duration of the follow-up period (table 2).

Maternal pH, P_a_CO_2_, P_a_O_2_, haemoglobin and arterial glucose concentration remained stable in the immediate post-operative period and for the duration of the follow-up period. There was a transient elevation of arterial lactate in both groups, which had resolved by the second post-operative day (table 2).

### 3.9. Maternal and fetal iatrogenic harm

**Figure 7.**
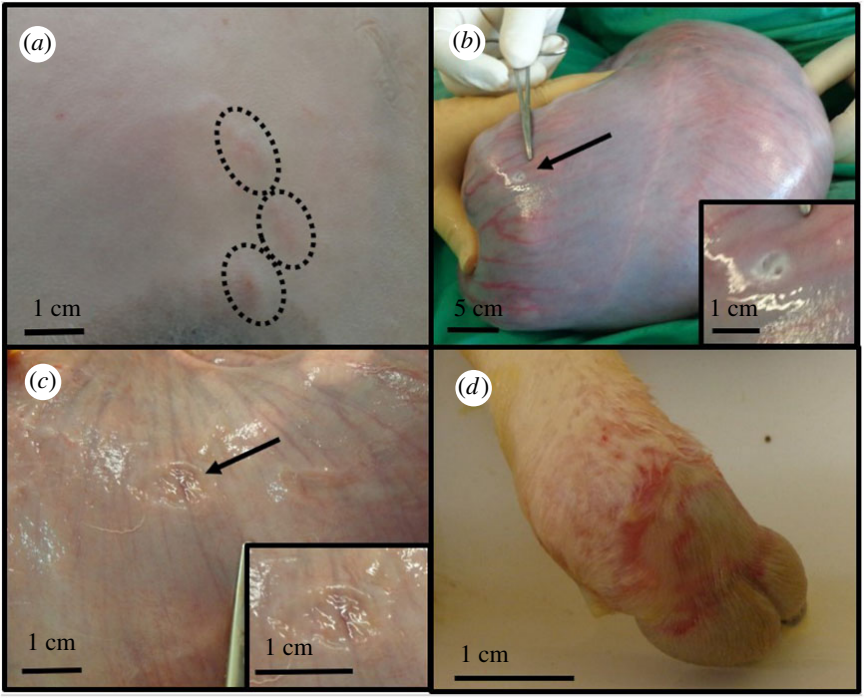
Maternal and fetal iatrogenic injury. (*a*) The photograph shows the maternal abdomen after shaving and depilation and exposure to six HIFU series. The circled areas are areas of skin redness, which appear to be erythema. (*b*) The photograph shows a serosal layer uterine burn immediately following HIFU exposures (noted at subsequent laparotomy-specific HIFU exposure series unknown). The inset shows a close-up of the affected area. (*c*) The photograph shows the same uterine burn after 21 days, demonstrating healing without rupture; the inset shows a close-up of the damage. (*d*) The photograph shows a skin burn on the anterior aspect of the left fetal hindlimb. This resulted from five HIFU exposures of estimated *in situ* *I*_SPTA_ 2.4 kW cm^−2^. (Online version in colour.)

There were no maternal skin burns as a result of the HIFU exposure series performed. In one sheep, erythema of the maternal abdominal skin was noted. In this animal, six HIFU exposure series were performed, and in five of six HIFU exposure series, maternal skin erythema resulted (figure 7*a*). These areas of skin redness were fully resolved by the time of anaesthetic reversal. The five HIFU exposure series in this animal which caused the skin erythema each delivered six HIFU exposures, 2 mm apart, with an estimated *in situ* intensity of 2.5–2.6 kW cm^−2^, to vascular targets 17–32 mm beneath the maternal skin. There was only one HIFU exposure series delivered to each target (which were all successfully occluded). No errors in targeting the blood vessels or operation of the HIFU system were identified. The sixth HIFU exposure series, which did not result in erythema, delivered seven exposures of 2.5 kW cm^−2^ at a target depth of 26 mm (electronic supplementary material, table S1). As such, there was no clear difference between the five exposure series that caused erythema and the one that did not, nor was there an obvious cause for the erythema based on operator or technical error. No maternal veterinary skin abnormality was noted, and there was no deviation from the standard method used for the preparation of the skin in this animal relative to the other ewes in this study. The maternal weight was 54 kg. There was no maternal skin erythema observed in the other five sheep exposed to HIFU.

There was a single instance of damage to the uterine serosa noted at the time of laparotomy (day 0). This area was not excised or repaired and had healed by the time of post-mortem examination (day 20). There was no evidence of uterine perforation or leakage of amniotic fluid resulting from this injury (figure 7*b,c*). It was not possible to determine which HIFU exposure series resulted in this uterine burn. The HIFU exposure series delivered in this animal, one of which caused the burn, were comprised of five to six exposures, delivering an estimated *in situ* intensity of 2.0–3.1 kW cm^−2^, to vascular targets 19–28 mm beneath the surface of the maternal skin. Only a single HIFU exposure series was delivered to each vascular target (all of which were occluded) and no errors in the operation of the HIFU system or in the targeting of the blood vessel were identified (electronic supplementary material, table S1). There were no maternal skin burns, erythema or fetal injury noted in this sheep. The maternal weight was 55 kg. Again, there was no obvious cause for the uterine burn.

There was a single instance of a burn to a fetal hind limb, which was first noted at the time of surgery during insertion of fetal catheters (day 0). This was a direct result of a targeting error and resulted in the failure of vascular occlusion, requiring the vessel to be retreated. The first (mistargeted) HIFU exposure series delivered five HIFU exposures of 2.4 kW cm^−2^ estimated *in situ* intensity to a depth of 20 mm beneath the maternal skin. The fetal hind limb was 29 mm beneath the surface of the skin and 9 mm beneath the vascular target, measured parallel to the direction of the sound. The second HIFU exposure series delivered six HIFU exposures of 2.6 kW cm^−2^ estimated *in situ* intensity to a depth of 22 mm beneath the maternal skin, after the fetal hind limb had spontaneously moved away, and resulted in vascular occlusion (electronic supplementary material, table S1). This injury remained evident at post-mortem (day 20) and histological examination suggested a full thickness skin burn (figure 7*d*). There was no evidence of anaemia or acidosis in this fetus during the follow-up period. There were no maternal uterine or skin burns, or erythema, in this animal. The maternal weight was 53 kg. This burn can be attributed to operator error and inadvertent failure to follow the safety constraints used for target selection and described in the Material and methods section.

## 4. Discussion

This study demonstrates non-invasive HIFU-mediated placental vascular occlusion with an efficacy of 100%, with no detrimental effect on maternal and fetal survival to term. Mothers and fetuses both made a full recovery from general anaesthesia, abdominal and fetal surgery, with (*n* = 6) or without (*n* = 6) exposure to HIFU placental vascular occlusion.

As in our previous study of the acute effects of general anaesthesia, abdominal and fetal surgery, with or without exposure to HIFU placental vascular occlusion [5], there were changes in fetal physiology by the end of the experimental protocol. In this study, following reversal of anaesthesia in both treatment groups, there was activation of the fetal sympathetic nervous system, a partially buffered mixed respiratory and metabolic acidosis, and a lowering of fetal oxyhaemoglobin saturation, but not of partial pressure of arterial oxygen.

Fetal sympathetic activation can be inferred from the increase in fetal peripheral vascular resistance [20] and fetal haemoconcentration [21] during the first 48 h following reversal of anaesthesia. Sympathetic stimulation of the splenic circulation and venous reservoirs decreases venous capacitance and acutely increases the red blood cell count within the circulation in the fetus [21], presenting as an increase in haemoglobin concentration and haematocrit, as seen in this study. Activation of the fetal sympathetic nervous system is further supported by the finding of an increase in the indices of fetal heart rate variability (FHRV) for the first 72 h following reversal of anaesthesia, attributable, in our results, to an increase in normalized LF rather than HF power. The fetal heart rate is known to be influenced by the sympathetic and parasympathetic nervous systems, both of which contribute to overall FHRV. Power spectral analysis of the fetal heart rate on a beat-to-beat basis allows assessment of the separate contributions of the sympathetic and parasympathetic nervous systems to overall FHRV. nLF power provides the contribution of the sympathetic nervous system and nHF power provides the equivalent information of the parasympathetic nervous system contribution [22,23]. In this study, there was an elevation in nLF power in both treatment groups which persisted for 72 h following the reversal in anaesthesia, which resulted in an increase in the indices of overall FHRV, STV and SDNN. There was no corresponding increase in nHF power.

It should be noted that this increased fetal sympathetic outflow appears to be the result of the surgical and experimental procedures described, but not of an intrauterine hypoxic insult, since the partial pressure of arterial oxygen remained within normal parameters at all times. This is supported by the mild increase in femoral arterial vascular resistance, which was, at its peak, only approximately 50% above baseline. In comparison, acute hypoxic insults which significantly lower fetal oxygenation have been reported to increase fetal femoral arterial resistance by between 300 and 400% [20]. Further, the sympathetically mediated haemoconcentration will contribute to the maintenance of oxygen delivery to the fetal tissues. Finally, increased FHRV in acute hypoxia has been shown to result from the activation of the parasympathetic rather than the sympathetic nervous system, and parasympathetic activation was not seen in our fetuses.

The fall in fetal pH can be attributed to both the accumulation of arterial carbon dioxide in the maternal and consequently the fetal circulations and to the accumulation of lactate in the fetal tissues. These are recognized effects of isoflurane general anaesthesia regardless of concomitant surgical or experimental procedures, which would not be required in any potential human treatment [24,25]. The redistribution of blood away from peripheral circulation results in anaerobic metabolism of glucose in peripheral tissues, further lowering fetal pH and thereby facilitating increased unloading of oxygen from haemoglobin to fetal tissues [26,27]. While the degree of acidosis in the fetus in both HIFU-exposed and control groups was comparable, there were differences observed in ABE and bicarbonate concentrations. In the HIFU-exposed group, the ABE was negative, and the bicarbonate concentrations were normal; in the control group, the ABE and bicarbonate were elevated. This suggests that the effect of respiratory acidosis was predominant in control animals, compared to HIFU-exposed animals, where metabolic acidosis was more apparent. This may have been related to the difference in maternal anaesthesia—ewes exposed to HIFU underwent periods of hyperventilation around HIFU exposures that were not replicated in control animals. However, despite these appropriate fetal metabolic compensatory responses to the surgical and experimental procedures experienced, physiological levels of oxygen and glucose were available at all points of the post-operative recovery and follow-up periods [28].

The recovery period for the fetal mixed acidosis, haemoconcentration and peripheral vasoconstriction was less than 48 h, with no difference between HIFU-exposed and control groups. Thereafter, fetal values remained within normal ranges corrected for gestational age [11,28,29]. Similarly, normalized LF power had returned to baseline levels within 72 h, as had the indices of overall FHRV. During the follow-up period, treatment had no effect on any measure of FHRV, in either quiet or active sleep states. Furthermore, the observation of expected ontogenic changes in fetal heart rate, blood pressure and arterial flows [11,30,31] is suggestive of the normal ongoing development of the fetal cardiovascular and endocrine system during this recovery period. This is supported by plasma cortisol levels, measured on 5 and 20 post-operative days, which showed increases with advancing gestational age but no difference between treatment groups. At 121 ± 2 days gestational age (post-operative day 5), fetal plasma cortisol levels should be around 10 ng ml^−1^, as in our animals, as the cortisol surge that matures fetal tissues and triggers parturition in sheep has yet to occur. After a further 15 days follow-up, fetal plasma cortisol levels were higher, as expected in the preparation for delivery, but not different between treatment groups [32]. This rise in endogenous cortisol levels approaching term underlies the increase in arterial blood pressure and arterial blood flow in sheep fetuses as the autonomic nervous system matures and begins to establish a resting tone [33,34]. Finally, normal fetal development is further supported by the comparable birth weights and body mass indices, without evidence of asymmetric growth, as previously reported [10]. As only singleton fetuses were used, these results are free of the potential confounding factors found in multiple pregnancies.

The HIFU protocol used in this study resulted in a high occlusion rate of placental vasculature, through intact maternal skin, despite a reduction in estimated *in situ* intensity delivered to the target tissue compared to our previous transdermal experience: from 2.9 (IQR 0.6) kW cm^−2^ [10] to 2.6 (IQR 0.4) kW cm^−2^. This change, together with cooling and continuous degassing of fluid in the water bag, was sufficient to reduce the rate of skin burns to zero compared to 11% (4/36) [10] in our previous transdermal study. The mean target depth was 24 mm, which is comparable to the distance which an anterior placenta lies beneath the abdominal skin in human pregnancy, meaning that the HIFU system used here is a clinically relevant one in terms of frequency used, lesion size and focal length.

The study protocol also demonstrated the ability to identify, target and use HIFU to occlude six placental vessels within 1 h. Recent work describing updates to fetoscopic laser procedures describes that a mean of 4 AVAs (range 1–11) need to be occluded in human treatment [35]. The use of a mechanical gantry to control the HIFU transducer means that the HIFU system can either be used for selective coagulation of AVAs or to emulate the Solomon technique, ablating soft tissue between anastomoses, although this has yet to be shown to reduce perinatal mortality or severe neonatal morbidity [36]. Both these features show the clinical relevance of the HIFU protocol and system used here. As both the identification of AVAs [37] and the vascular equator of the placenta [38] using ultrasound have been described, this would not be expected to become a barrier to translation.

Similarly, the pregnant sheep model is a good anatomical model of the anastomoses within the human monochorionic placenta, which does not alter with the gestational age of the sheep pregnancy, and was the first model in which fetoscopic laser was tested [39]. The sheep placental mass is divided into 70–80 areas of enmeshed maternal and fetal tissue and villi, called placentomes, where haemotrophic exchange takes place. Fetal vessels emerge from placentomes and run between placentomes in the allantoic membranes and can therefore be targeted within placental tissue as they would need to be in a monochorionic placenta. In monochorionic placentae, arterio-venous anastomoses are reported to be 0.3–2.1 mm in diameter [40,41]. The vessels occluded in our study had a mean diameter of 1.4 mm, with a maximum diameter of up to 4.1 mm, demonstrating the capability of the HIFU system used to occlude vessels of a clinically relevant size.

The most significant injury encountered in this study was a burn to a fetal hind limb. As detailed in the Results section, this resulted from both mistargeting, and the fetal hind limb moving closer to the target between mapping and delivery of HIFU, without being noted. While undesirable, this event illustrates several points. Firstly, it shows the importance of continuously identifying the fetal position relative to the HIFU focal zone, using real-time imaging techniques. Secondly, it emphasizes the importance of building in to the HIFU protocol systematic safety checks to reduce the risk of unwanted effects that can reasonably be anticipated. Thirdly, it is reassuring that this fetus recovered without evidence of compromise, despite the burn. This is an important finding when considering translation into human therapy.

Additionally, there was a single uterine serosal burn, the cause of which was not established. There was nothing unusual about the target vessels in terms of size, type or placement of vessel which could explain the burn. While the maternal weight—55 kg—was at the upper end of the range of weights recorded, three other ewes with weights 53–55 kg did not sustain uterine burns. Regardless of the cause, this injury did not lead to uterine rupture or other obstetric complications.

In conclusion, maternal and fetal recovery from HIFU-mediated placental vascular occlusion, followed by normal long-term normal physiology is possible. We observed no evidence of fetal death, compromise or stress, while demonstrating normal maturation and development the fetal cardiovascular and autonomic nervous systems. This study also demonstrated that such positive results can be achieved in the presence of uterine and fetal burns. Combined with the high success rate of selective placental vascular occlusion, HIFU has potential as an *in utero* therapy in relevant human pregnancy conditions, such as TTTS where highly invasive therapy is otherwise currently the only option.

## Supplementary Material

Table s1

## Supplementary Material

Figure s1: Timing and frequency of monitoring during follow-up period

## Supplementary Material

Figure s2: HIFU therapy system setup.

## Data accessibility

All relevant data are presented within the paper, its electronic supplementary material files or can be accessed via the Open Science Framework: https://osf.io/wk96v.

## References

[1] Shaw CJ, ter Haar GR, Rivens IH, Giussani DA, Lees CC 2014 Pathophysiological mechanisms of high-intensity focused ultrasound-mediated vascular occlusion and relevance to non-invasive fetal surgery. J. R Soc. Interface 11, 20140029 (10.1098/rsif.2014.0029) 24671935 PMC4006242

[2] Okai T et al*.* 2013 First successful case of non-invasive in-utero treatment of twin reversed arterial perfusion sequence by high-intensity focused ultrasound. Ultrasound Obstet. Gynecol. 42, 112–114. (10.1002/uog.12466) 23533101

[3] Seo K et al*.* In press. Twin-reversed arterial perfusion sequence using high-intensity focused ultrasound therapy. Ultrasound Obstet. Gynecol. (10.1002/uog.20101) 30136326

[4] Caloone J et al*.* 2015 High-intensity focused ultrasound applied to the placenta using a toroidal transducer: a preliminary ex-vivo study. Ultrasound Obstet. Gynecol. 45, 313–319. (10.1002/uog.13374) 24723334

[5] Shaw CJ et al 2016 Noninvasive high-intensity focused ultrasound treatment of twin-twin transfusion syndrome: a preliminary *in vivo* study. Sci. Transl. Med. 8, 347ra395. 10.1126/scitranslmed.aaf2135 27412787

[6] Roberts D, Neilson JP, Kilby MD, Gates S 2014 Interventions for the treatment of twin-twin transfusion syndrome. Cochrane Database Syst. Rev. 1, CD002073. 10.1002/14651858.CD002073.pub3 PMC10816955 24482008

[7] Ichizuka K, Hasegawa J, Nakamura M, Matsuoka R, Sekizawa A, Okai T, Umemura S 2012 High-intensity focused ultrasound treatment for twin reversed arterial perfusion sequence. Ultrasound Obstet. Gynecol. 40, 476–478. (10.1002/uog.11114) 22302667

[8] Kim Y et al*.* 2011 Non-invasive pulsed cavitational ultrasound for fetal tissue ablation: feasibility study in a fetal sheep model. Ultrasound Obstet. Gynecol. 37, 450–457. (10.1002/uog.8880) 21433165

[9] Paek BW, Vaezy S, Fujimoto V, Bailey M, Albanese CT, Harrison MR, Farmer DL 2003 Tissue ablation using high-intensity focused ultrasound in the fetal sheep model: potential for fetal treatment. Am. J. Obstet. Gynecol. 189, 702–705. (10.1067/S0002-9378(03)00664-1) 14526297

[10] Shaw CJ, Rivens I, Civale J, Botting KJ, Ter Haar G, Giussani DA, Lees CC 2018 Trans-abdominal in vivo placental vessel occlusion using high intensity focused ultrasound. Sci. Rep. 8, 13631 (10.1038/s41598-018-31914-4) 30206278 PMC6134117

[11] Allison BJ et al 2016 Fetal in vivo continuous cardiovascular function during chronic hypoxia. J. Physiol. 594, 1247–1264. (10.1113/JP271091) 26926316 PMC4771786

[12] Shaw CJ 2018 Developing a non-invasive treatment for twin-twin transfusion syndrome using high intensity focused ultrasound in an animal model. PhD thesis, Imperial College London, London, UK Appendix IV.

[13] Brain KL, Allison BJ, Niu Y, Cross CM, Itani N, Kane AD, Herrera EA, Giussani DA 2015 Induction of controlled hypoxic pregnancy in large mammalian species. Physiol. Rep. 3, e12614 (10.14814/phy2.12614) 26660546 PMC4760453

[14] Street P, Dawes GS, Moulden M, Redman CW 1991 Short-term variation in abnormal antenatal fetal heart rate records. Am. J. Obstet. Gynecol. 165, 515–523. (10.1016/0002-9378(91)90277-X) 1892175

[15] Shaw CJ et al 2018 Altered autonomic control of heart rate variability in the chronically hypoxic fetus. J. Physiol. 596, 6105–6119. (10.1113/JP275659) 29604064 PMC6265555

[16] Min SW, Ko H, Kim CS 2002 Power spectral analysis of heart rate variability during acute hypoxia in fetal lambs. Acta Obstet. Gynecol. Scand. 81, 1001–1005. (10.1034/j.1600-0412.2002.811102.x) 12421166

[17] van Laar JO, Peters CH, Vullings R, Houterman S, Oei SG. 2009 Power spectrum analysis of fetal heart rate variability at near term and post term gestation during active sleep and quiet sleep. Early Hum. Dev. 85, 795–798. (10.1016/j.earlhumdev.2009.11.001) 19931326

[18] Koome ME, Bennet L, Booth LC, Wassink G, Davidson JO, Gunning M, Gunn AJ 2014 Quantifying the power spectrum of fetal heart rate variability. Exp. Physiol. 99, 468 (10.1113/expphysiol.2013.077123) 24487250

[19] Lear CA et al 2016 Sympathetic neural activation does not mediate heart rate variability during repeated brief umbilical cord occlusions in near-term fetal sheep. J. Physiol. 594, 1265–1277. (10.1113/JP270125) 25864517 PMC4771778

[20] Thakor AS, Allison BJ, Niu Y, Botting KJ, Herrera EA, Giussani DA 2015 Melatonin modulates the fetal cardiovascular defense response to acute hypoxia. J. Pineal Res. 59, 80–90. (10.1111/jpi.12242) 25908097 PMC4528231

[21] Potocnik SJ, Wintour EM 1996 Development of the spleen as a red blood cell reservoir in lambs. Reprod. Fertil. Dev. 8, 311–315. (10.1071/RD9960311) 8795091

[22] Kimura Y, Andersen TT, Fenton JW, Bahou WF, Aviv A 1996 Power spectral analysis for autonomic influences in heart rate and blood pressure variability in fetal lambs. Am. J. Physiol. 271, 1333–1339. (10.1152/ajpcell.1996.271.1.C54) 8897925

[23] Akselrod S, Gordon D, Ubel F, Shannon D, Berger A, Cohen R 1981 Power spectrum analysis of heart rate fluctuation: a quantitative probe of beat-to-beat cardiovascular control. Science 213, 220–222. (10.1126/science.6166045) 6166045

[24] McClaine RJ et al*.* 2005 General anesthesia improves fetal cerebral oxygenation without evidence of subsequent neuronal injury. J. Cereb. Blood Flow Metab. 25, 1060–1069. (10.1038/sj.jcbfm.9600094) 15758947

[25] McClaine RJ et al 2007 A description of the preterm fetal sheep systemic and central responses to maternal general anesthesia. Anesth. Analg. 104, 397–406. (10.1213/01.ane.0000252459.43933.59) 17242098

[26] Boyle DW, Meschia G, Wilkening RB 1992 Metabolic adaptation of fetal hindlimb to severe, nonlethal hypoxia. Am. J. Physiol. 263, R1130–R1135. 1443231 10.1152/ajpregu.1992.263.5.R1130

[27] Gardner DS, Giussani DA 2003 Enhanced umbilical blood flow during acute hypoxemia after chronic umbilical cord compression: a role for nitric oxide. Circulation 108, 331–335. (10.1161/01.CIR.0000080323.40820.A1) 12835209

[28] Jellyman JK, Gardner DS, Edwards CM, Fowden AL, Giussani DA 2005 Fetal cardiovascular, metabolic and endocrine responses to acute hypoxaemia during and following maternal treatment with dexamethasone in sheep. J. Physiol. 567, 673–688. (10.1113/jphysiol.2005.089805) 15975982 PMC1474208

[29] Fletcher AJ, Gardner DS, Edwards CM, Fowden AL, Giussani DA 2003 Cardiovascular and endocrine responses to acute hypoxaemia during and following dexamethasone infusion in the ovine fetus. J. Physiol. 549, 271–287. (10.1113/jphysiol.2002.036418) 12665612 PMC2342926

[30] Fletcher AJ, Gardner DS, Edwards CM, Fowden AL, Giussani DA 2006 Development of the ovine fetal cardiovascular defense to hypoxemia towards full term. Am. J. Physiol. Heart Circ. Physiol. 291, H3023–H3034. (10.1152/ajpheart.00504.2006) 16861695

[31] Giussani DA, Forhead AJ, Fowden AL 2005 Development of cardiovascular function in the horse fetus. J. Physiol. 565, 1019–1030. (10.1113/jphysiol.2004.078469) 15790668 PMC1464542

[32] Forhead AJ, Fowden AL 2004 Role of angiotensin in the pressor response to cortisol in fetal sheep during late gestation. Exp. Physiol. 89, 323–329. (10.1113/expphysiol.2004.027185) 15123568

[33] Tangalakis K, Lumbers ER, Moritz KM, Towstoless MK, Wintour EM 1992 Effect of cortisol on blood pressure and vascular reactivity in the ovine fetus. Exp. Physiol. 77, 709–717. (10.1113/expphysiol.1992.sp003637) 1418954

[34] Derks JB, Giussani DA, Jenkins SL, Wentworth RA, Visser GH, Padbury JF, Nathanielsz PW 1997 A comparative study of cardiovascular, endocrine and behavioural effects of betamethasone and dexamethasone administration to fetal sheep. J. Physiol. 499, 217–226. (10.1113/jphysiol.1997.sp021922) 9061651 PMC1159348

[35] Ierullo AM, Papageorghiou AT, Bhide A, Fratelli N, Thilaganathan B 2007 Severe twin-twin transfusion syndrome: outcome after fetoscopic laser ablation of the placental vascular equator. Br. J. Obstet. Gynecol. 114, 689–693. (10.1111/j.1471-0528.2007.01336.x) 17516959

[36] Slaghekke F et al*.* 2014 Fetoscopic laser coagulation of the vascular equator versus selective coagulation for twin-to-twin transfusion syndrome: an open-label randomised controlled trial. Lancet 383, 2144–2151. (10.1016/S0140-6736(13)62419-8) 24613024

[37] Taylor MJ, Farquharson D, Cox PM, Fisk NM 2000 Identification of arterio-venous anastomoses in vivo in monochorionic twin pregnancies: preliminary report. Ultrasound Obstet. Gynecol. 16, 218–222. (10.1046/j.1469-0705.2000.00227.x) 11169285

[38] Quintero RA et al 2005 Individual placental territories after selective laser photocoagulation of communicating vessels in twin-twin transfusion syndrome. Am. J. Obstet. Gynecol. 192, 1112–1118. (10.1016/j.ajog.2004.12.018) 15846189

[39] De Lia JE, Cruikshank DP, Keye WR Jr 1990 Fetoscopic neodymium: YAG laser occlusion of placental vessels in severe twin-twin. Obstet. Gynecol. 75, 1046–1053. (10.1016/0020-7292(91)90254-3) 2342732

[40] Zhao DP, De Villiers SF, Slaghekke F, Walther FJ, Middeldorp JM, Oepkes D, Lopriore E 2013 Prevalence, size, number and localization of vascular anastomoses in monochorionic placentas. Placenta 34, 589–593. (10.1016/j.placenta.2013.04.005) 23639577

[41] Lewi L et al*.* 2006 Intertwin anastomoses in monochorionic placentas after fetoscopic laser coagulation for twin-to-twin transfusion syndrome: is there more than meets the eye? Am. J. Obstet. Gynecol. 194, 790–795. (10.1016/j.ajog.2005.08.062) 16522414

