# Supplementary material for: Maternal and fetal cardiometabolic recovery following ultrasound-guided high-intensity focused ultrasound placental vascular occlusion
Source: J R Soc Interface. 2019 May 1;16(154):20190013. doi: 10.1098/rsif.2019.0013 (PMC6544891; doi:10.1098/rsif.2019.0013)
Supplement: Table s1 [file rsif20190013supp1.docx]

**Electronic Supplementary Material**

**Journal of the Royal Society Interface**

# Maternal and fetal cardiometabolic recovery following ultrasound guided high intensity focused ultrasound (HIFU) placental vascular occlusion

Caroline J. Shaw, Ian Rivens, John Civale, Kimberley J. Botting, Beth J. Allison, Kirsty L. Brain, Y. Niu, Gail ter Haar, Dino A. Giussani, Christoph C. Lees

**Table s1:** Exposure characteristics and outcomes of HIFU exposure series categorised by animal and vascular target.
