# Supplementary material for: Maternal and fetal cardiometabolic recovery following ultrasound-guided high-intensity focused ultrasound placental vascular occlusion
Source: J R Soc Interface. 2019 May 1;16(154):20190013. doi: 10.1098/rsif.2019.0013 (PMC6544891; doi:10.1098/rsif.2019.0013)
Supplement: Figure s1: Timing and frequency of monitoring during follow-up period [file rsif20190013supp2.docx]

**Electronic Supplementary Material**

**Journal of the Royal Society Interface**

# Maternal and fetal cardiometabolic recovery following ultrasound guided high intensity focused ultrasound (HIFU) placental vascular occlusion

Caroline J. Shaw, Ian Rivens, John Civale, Kimberley J. Botting, Beth J. Allison, Kirsty L. Brain, Y. Niu, Gail ter Haar, Dino A. Giussani, Christoph C. Lees


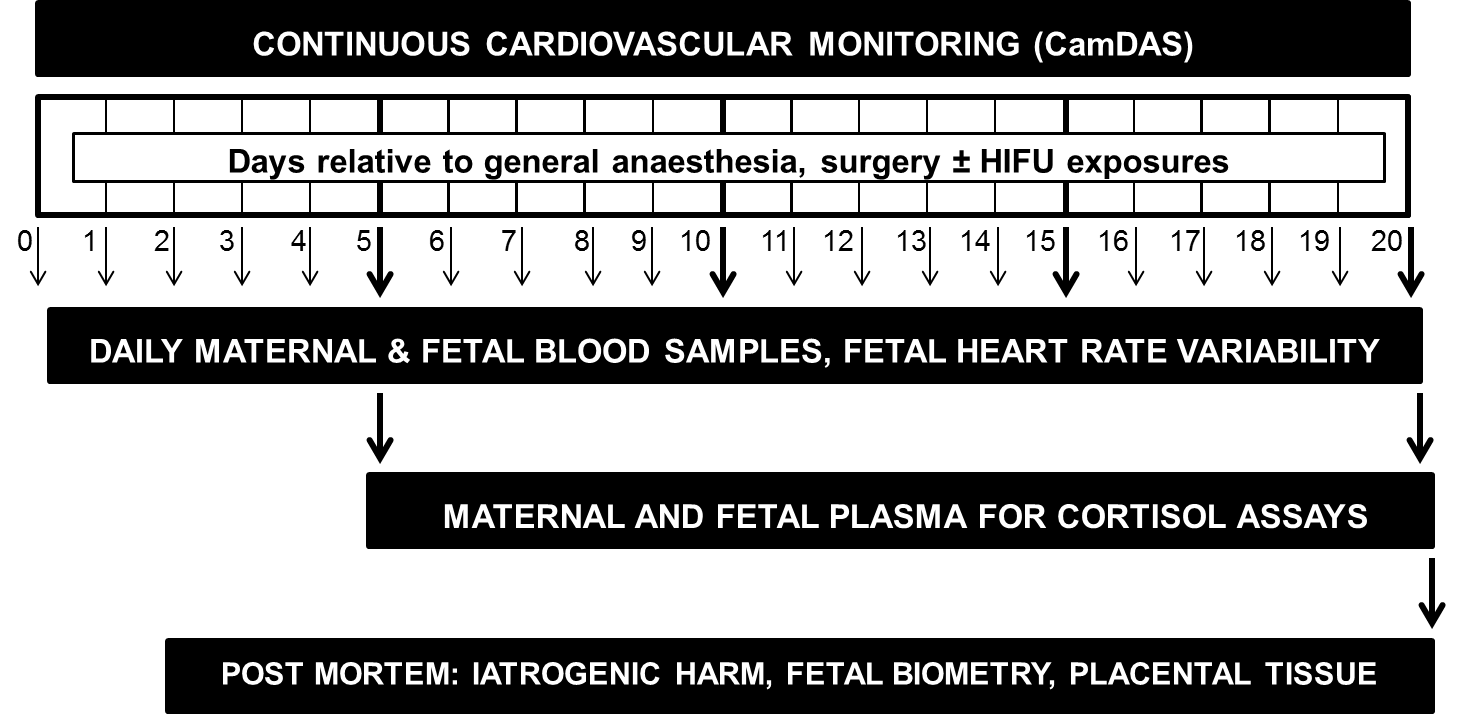


Figure s1: Timing and frequency of monitoring during follow-up period

The schema shows the relative frequency and timing of cardiovascular monitoring, blood sampling for metabolic and endocrine analysis, sampling of the fetal heart rate for analysis of variability, and post mortem examination.
