# Supplementary material for: Maternal and fetal cardiometabolic recovery following ultrasound-guided high-intensity focused ultrasound placental vascular occlusion
Source: J R Soc Interface. 2019 May 1;16(154):20190013. doi: 10.1098/rsif.2019.0013 (PMC6544891; doi:10.1098/rsif.2019.0013)
Supplement: Figure s2: HIFU therapy system setup. [file rsif20190013supp3.docx]

**Electronic Supplementary Material**

**Journal of the Royal Society Interface**

# Maternal and fetal cardiometabolic recovery following ultrasound guided high intensity focused ultrasound (HIFU) placental vascular occlusion

Caroline J. Shaw, Ian Rivens, John Civale, Kimberley J. Botting, Beth J. Allison, Kirsty L. Brain, Y. Niu, Gail ter Haar, Dino A. Giussani, Christoph C. Lees


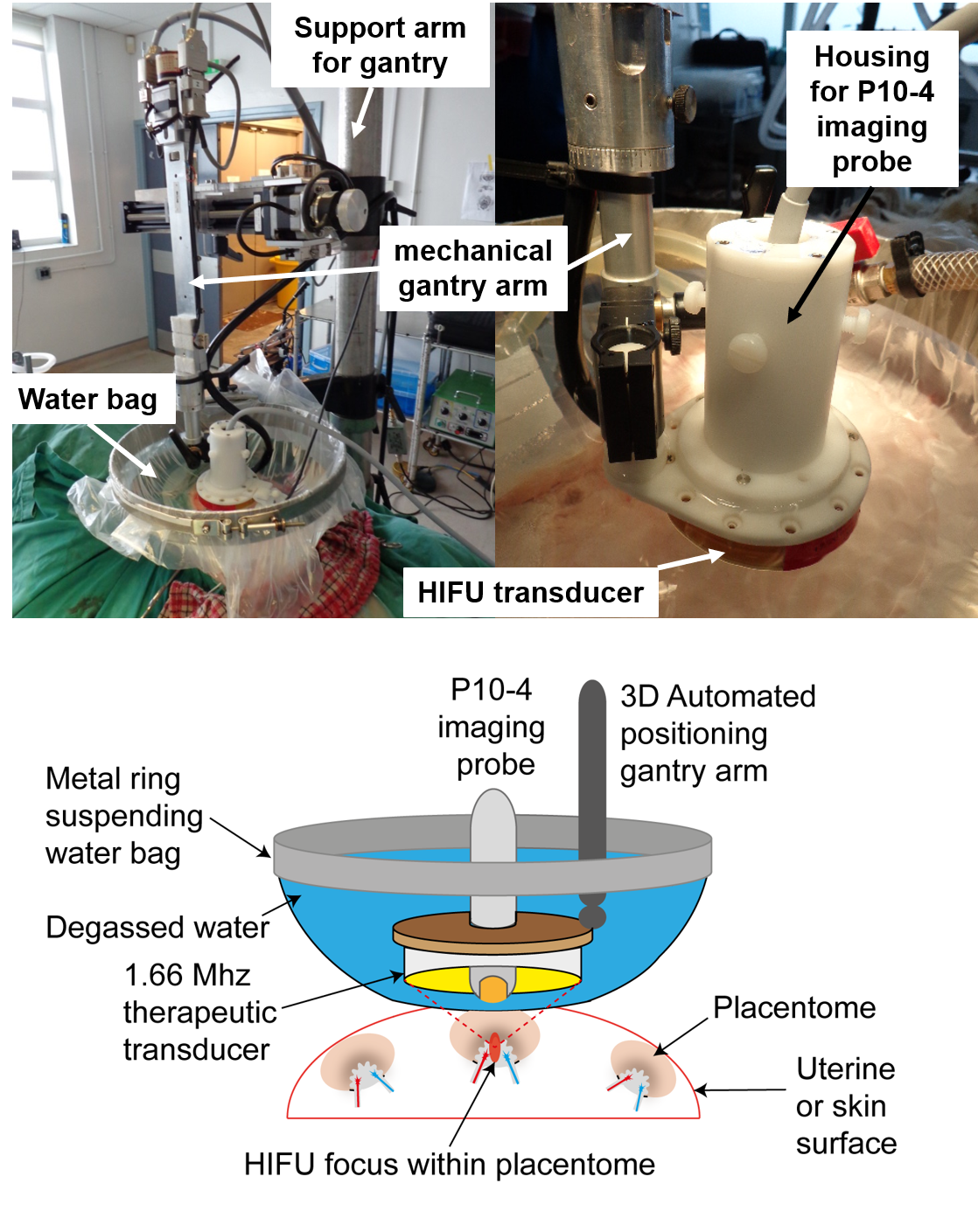


**Figure s2: HIFU therapy system setup.**

Top left: the picture shows the mechanical gantry arm and associated support structures in relationship to the water bag, HIFU and diagnostic ultrasound transducers. The ewe is lying supine, covered by surgical drapes (green), with maternal abdominal skin exposed only beneath the water bag. Top right: the picture shows an enlargement of the HIFU therapy transducer and the housing of the diagnostic transducer which allow integration of the two transducers, and their attachment to the mechanical gantry arm. Bottom: the diagram shows the arrangement of diagnostic and HIFU transducers in relationship to the gantry arm, the water bag, degassed water, and the maternal uterine or skin surface (not to scale).
